# Low Mutation Burden in Ovarian Cancer May Limit the Utility of Neoantigen-Targeted Vaccines

**DOI:** 10.1371/journal.pone.0155189

**Published:** 2016-05-18

**Authors:** Spencer D. Martin, Scott D. Brown, Darin A. Wick, Julie S. Nielsen, David R. Kroeger, Kwame Twumasi-Boateng, Robert A. Holt, Brad H. Nelson

**Affiliations:** 1 Trev and Joyce Deeley Research Centre, British Columbia Cancer Agency, Victoria, Canada; 2 Interdisciplinary Oncology Program, University of British Columbia, Vancouver, Canada; 3 Michael Smith’s Genome Sciences Centre, British Columbia Cancer Agency, Vancouver, Canada; 4 Molecular Biology and Biochemistry, Simon Fraser University, Vancouver, Canada; 5 Department of Medical Genetics, University of British Columbia, Vancouver, Canada; 6 Department of Microbiology and Biochemistry, University of Victoria, Victoria, Canada; McMaster University, CANADA

## Abstract

Due to advances in sequencing technology, somatically mutated cancer antigens, or neoantigens, are now readily identifiable and have become compelling targets for immunotherapy. In particular, neoantigen-targeted vaccines have shown promise in several pre-clinical and clinical studies. However, to date, neoantigen-targeted vaccine studies have involved tumors with exceptionally high mutation burdens. It remains unclear whether neoantigen-targeted vaccines will be broadly applicable to cancers with intermediate to low mutation burdens, such as ovarian cancer. To address this, we assessed whether a derivative of the murine ovarian tumor model ID8 could be targeted with neoantigen vaccines. We performed whole exome and transcriptome sequencing on ID8-G7 cells. We identified 92 somatic mutations, 39 of which were transcribed, missense mutations. For the 17 top predicted MHC class I binding mutations, we immunized mice subcutaneously with synthetic long peptide vaccines encoding the relevant mutation. Seven of 17 vaccines induced robust mutation-specific CD4 and/or CD8 T cell responses. However, none of the vaccines prolonged survival of tumor-bearing mice in either the prophylactic or therapeutic setting. Moreover, none of the neoantigen-specific T cell lines recognized ID8-G7 tumor cells *in vitro*, indicating that the corresponding mutations did not give rise to *bonafide* MHC-presented epitopes. Additionally, bioinformatic analysis of The Cancer Genome Atlas data revealed that only 12% (26/220) of HGSC cases had a ≥90% likelihood of harboring at least one authentic, naturally processed and presented neoantigen versus 51% (80/158) of lung cancers. Our findings highlight the limitations of applying neoantigen-targeted vaccines to tumor types with intermediate/low mutation burdens.

## Introduction

Somatically mutated cancer antigens, or “neoantigens”, are attractive immunotherapy targets that have recently become accessible due to advances in next generation sequencing (NGS) technologies[1,2]. Unlike cancer/testes (CT) or differentiation antigens, which are encoded in the germ line, neoantigens are tumor restricted and are not expressed in the thymus or other non-malignant tissues. Therefore, high-affinity neoantigen-reactive T cells escape negative selection in the thymus, and on-target/off-tumor toxicities are minimized. The contribution of neoantigens to successful immunotherapy is becoming increasingly evident. Clinical responses to anti-PD-1[3] and -CTLA-4[4,5] antibodies have been associated with high mutation load, suggesting that neoantigens may be the most relevant target antigens underlying successful immune checkpoint blockade. Moreover, there is increasing evidence that neoantigen-specific T cells frequently underlie successful therapy with tumor-infiltrating lymphocytes (TIL)[6]. In the first published clinical study to deliberately target an NGS-identified neoantigen, adoptive transfer of a near-clonal population of neoantigen-reactive T cells resulted in regression of a metastatic cholangiocarcinoma[7]. However, since the vast majority of mutations, and hence neoantigens, are unique to individual patients, therapeutic targeting of neoantigens requires an individualized approach[1,2,8–13]. Although this represented a major obstacle in the past, such approaches are increasingly feasible in the modern era of personalized oncology[14–16].

For a mutation to give rise to a mutant neoantigen, several criteria must be met: a) the mutation must be present within a peptide that is processed from the parent protein by intracellular antigen processing machinery; b) the mutant peptide must bind with sufficient affinity to MHC; and c) the patient’s immune repertoire must contain T cells with sufficient affinity and specificity for the mutant epitope. As a result of these criteria, only a small percentage of mutations give rise to authentic T cell epitopes. For example, *in silico* analysis of all possible 9mers from a set of viral proteomes revealed that a median of 2% (range of .07% to 10.4%) of peptides bind to a given HLA allele with an IC50 < 500 nM[17]. Furthermore, a second study of viral epitopes found that only 8% of peptides that bound to MHCI with an IC50 < 100 nM represented authentic epitopes, meaning that they were naturally processed, presented on MHCI, and recognized by autologous CD8 T cells[18]. From these data, one would predict that only a small proportion of mutations give rise to authentic neoantigens. Since the number of somatic point mutations in human tumors can vary by five orders of magnitude within and between tumor types[19,20], from the perspective of neoantigen load, some cancers are intrinsically more immunogenic than others. Indeed, bioinformatic analysis of The Cancer Genome Atlas (TCGA) data revealed that increased point mutation and neoantigen burdens are associated with increased cytotoxic T cell infiltration[21,22], underscoring the relationship between neoantigen load and immune recognition of tumors.

Several studies have used NGS data to systematically assess recognition of somatic point mutations by CD4 and CD8 TIL. Initial studies were conducted in melanoma patients who had responded to checkpoint blockade or adoptive T cell therapy. Anywhere from 0 to 3 neoantigens were recognized per patient[10,13,23,24]. Given that melanomas harbor a median of 130 non-synonymous point mutations[25], these findings suggest that only a minor proportion of mutations give rise to authentic neoantigens. Two studies of carcinomas have used NGS to identify neoantigen reactive T cells in a comprehensive manner prior to any immunotherapy being administered. Our group sequenced primary and recurrent tumors from three HGSC patients and identified a single neoantigen-reactive T cell clone in TIL from one patient; combining the data from all three patients indicates that 1.3% (1/79) of mutations gave rise to authentic MHCI epitopes[12]. Moreover, in a study of 10 gastrointestinal tumors harboring a combined total of 1,452 mutations, 18 mutations (1.2% of the total) were recognized by CD4 or CD8 TIL (range = 0–3 per case)[26]. Thus, these latter two studies indicate that about 1% of point mutations give rise to neoantigens that elicit spontaneous TIL responses.

Whereas the above studies were based on the analysis of pre-existing T cell responses, others have investigated the extent to which neoantigen-specific T cell responses can be primed through vaccination. Indeed, human vaccine studies demonstrated that T cell responses can be elicited against mutations in common driver genes, including point mutations in KRAS[27] and p53[28] and to breakpoint sequences in the fusion oncoprotein BCR-ABL[29]. With NGS, one can expand this approach beyond established, shared mutations to the entire complement of somatic mutations in an individual’s tumor (referred to as the mutanome). By applying MHC binding algorithms to mutanome data, one can design personalized vaccines that target the best predicted mutant epitopes in a given tumor. Neoantigen-specific vaccines based on this approach have shown therapeutic efficacy in murine models of melanoma, carcinoma, and carcinogen-induced sarcoma[30–33]. However, to date, such pre-clinical studies have targeted tumors containing anywhere from 949 to 4285 nonsynonymous mutations[30–33]. In contrast, human tumors contain a median of only 44 non-synonymous mutations[20], raising the question of whether tumors with intermediate to low mutation burdens are amenable to neoantigen-targeted vaccination.

High grade serous carcinoma (HGSC) is the most common histological subtype of ovarian cancer, and long-term survival rates have not improved beyond 30–40% for the last 30 years[34]. Similar to other cancers, the presence of tumor-infiltrating T cells is strongly associated with survival in HGSC[35], which has inspired efforts to develop immune-based treatments for this disease[36]. Several candidate target antigens have been identified, including p53[37], WT-1[38], NY-ESO-1[39], mesothelin[40], and folate receptor[41]. However, these represent self antigens, which are subject to issues of immunological tolerance and autoreactivity. Furthermore, therapeutic targeting of these antigens using vaccines[37,38], or CAR T cells[42] has yielded only sporadic responses in HGSC to date. Thus, the possibility of treating HGSC with neoantigen-specific vaccines is compelling from both an immunological and clinical standpoint. However, in contrast to highly mutated malignancies such as melanoma, HGSC has been found to harbor a median of only 40 missense mutations per tumor[25], placing it very near the median for human cancers[20]. Thus, HGSC is an ideal disease for testing the applicability of neoantigen-targeted vaccines in malignancies with intermediate mutation loads.

To this end, we performed preclinical vaccine studies using a derivative of the murine ovarian cancer model ID8. The ID8 line has been used extensively to study the biological and immunological characteristics of ovarian cancer[43–45]. It arose by spontaneous transformation of normal murine ovarian surface epithelial (OSE) cells during serial *in vitro* passage[46]; therefore, it was not expected to harbor the high number of mutations found in carcinogen-induced tumors. Though most human HGSC are thought to arise from the distal fallopian tube secretory epithelial cells[34], the OSE-derived ID8 line gives rise to aggressive, widely disseminated cancers that are pathologically and histologically similar to human HGSC[43,46]. The line is sensitive to immune checkpoint blockade[45] and adoptive T cell therapy[47], making it an attractive system to assess new T cell-based immunotherapies. After performing whole exome and transcriptome sequencing on a derivative of the ID8 line, we systematically evaluated all identified nonsynonymous mutations as targets for prophylactic and therapeutic vaccination. We compared our findings to 220 human HGSC cases using data from The Cancer Genome Atlas (TCGA). Our findings indicate that the relatively low mutation load in both ID8 tumors and human HGSC presents a major challenge for neoantigen-based vaccines.

## Methods and Materials

### Exome and RNA sequencing

Library construction, sequencing, and bioinformatic analysis were performed at the Michael Smith Genome Sciences Centre. Genomic DNA was extracted from cultured ID8-G7 tumor cells and normal C57Bl/6 splenocytes. DNA was prepared for exome selection according to the manufacturer’s protocol using Agilent SureSelectXT Reagent kit HSQ (Cat# G9611A), and DNA was enriched for exomes using Agilent SureSelectXT All Mouse Exon Kit (Cat# 5190–4641). 100bp, paired-end sequencing was performed using the Illumina HiSeq2000 platform. After RNA extraction, mRNA was enriched using Miltenyi Biotec μMACS mRNA Isolation Kit (Cat# 130-090-276) and converted to cDNA using Invitrogen Superscript Double-Stranded cDNA Synthesis Kit (Cat# 11917–010). cDNA was sheared, end polished, and ligated to adapters followed by 100bp paired-end sequencing on the Illumina HiSeq2000 platform.

### Bioinformatic analysis

100bp paired-end reads from exome sequencing were mapped to the murine reference genome (mm9) using Burrows-Wheeler Aligner (BWA)[48]. Duplicates were flagged using Picard MarkDuplicates (version 1.102), and SNV and indels were identified using Samtools (version 0.1.18) pileup and VarFilter[49]. Variants were annotated using Annovar[50], and visually inspected using Integrated Genomics Viewer (IGV) (version 2.3.32)[51]. All variants found in dbSNP138 were removed. RNA-seq reads were aligned to a genome-plus-junctions reference (mm9) using BWA, and reads that mapped to junctions were repositioned as large-gapped genomic alignments. Reads were visualized using IGV to determine whether RNA sequencing reads contained mutated alleles identified in the exome sequencing data. Bam sequencing files are available at the NCBI sequence read archive with the following accession number: SRP069102. If both RNA and exome sequencing data contained a variant that was not present in the C57BL/6 exome sequencing data, the variant was deemed a tumor-specific mutation. The predicted binding affinity to H-2Kb and H-2Db of mutated peptides was determined using NetMHCpan2.4[52].

### Mice and tumor cell line

C57BL/6 mice were purchased from Jackson Labs and maintained under specific pathogen-free conditions. All mouse protocols were approved by the Animal Care Advisory Committee at the University of Victoria (permit number: 2011-032(2)), following Canadian Council for Animal Care guidelines. Adoptive cell transfer of splenocytes from female OT-I transgenic mice (Jackson Labs) containing T cells recognizing the chicken ovalbumin (OVA)_257-264_ epitope SIINFEKL were used as positive controls for tumor bearing experiments[53]. Mouse mammary tumor virus MMTV/*neu*^*OT-I/OT-II*^ transgenic mice were used as hosts in tumor-bearing experiments[54]. These mice are tolerant to the SIINFEKL epitope due to expression of the OT-I epitope (SIINFEKL) tagged onto the *neu* gene in mammary epithelial tissue. ID8 tumor cells[46] were generously donated by Drs. Paul Terranova and Katherine Roby in 2001 and modified in house to express the SIINFEKL epitope tagged to the rat *neu* oncogene. ID8-G7 tumor cells[47] were grown in High Glucose DMEM (Hyclone: SH30022.01) supplemented with 5% FBS (Gibco: 12483–020), 2mM L-glutamine (Hyclone: SH30034.01), 100U/ml Penicillin and 100μg/ml Streptomycin (Hyclone: SV30010), 50 μm β-mercaptoethanol (Sigma: M6250), and 1X Insulin Transferrin Selenium (ITS) (Corning: 354351).

### Vaccinations

Six to twelve week old female C57Bl/6 wild type (WT) or MMTV/*neu*^*OT-I/OT-II*^ mice were anesthetized and subcutaneously inoculated in the flank using a 27 gauge needle. Each inoculation was comprised of 25–50 μg of peptide (ProImmune) or 100 μg of chicken ovalbumin (OVA) (Sigma: cat# A5503) and 10 μg poly(I:C) (Amersham: 27–4732) admixed to a total of 300 μl in sterile PBS (Gibco: 20012–027). For therapeutic vaccination experiments, mice received daily vaccinations on days 3–6 days after tumor inoculation. For all other vaccination experiments, mice received daily vaccinations on days -28 to -25 and -7 to -4 with either tumor implantation or sacrifice of mice on day 0.

### Tumor-bearing mouse experiments

ID8-G7 tumor cells were detached from plates using trypsin, washed 3 times in sterile PBS, and re-suspended in sterile PBS at 10^7^ cells/ml. The cell suspension (10^6^ cells in 100μl) was injected into the peritoneal cavity of each mouse. Mice were monitored and euthanized at the first sign of abdominal distension due to ascites accumulation. On necropsy, all mice with abdominal distension contained highly disseminated tumors. For adoptive transfer experiments, OT-I transgenic mice were euthanized, and splenocytes were processed by pressing splenocytes through a 100μm nylon filter, lysing red blood cells with ACK lysis buffer (Lonza: 10-548E), and counting live splenocytes by trypan blue staining. OT-I splenocytes (10^5^ cells in 100 μl) were injected into the tail vein of recipient mice, followed by vaccination with 100 μg of OVA and 10μg of poly(I:C) on four consecutive days.

### IFN-γ and IL-2 ELISPOT assays

In accord with MIATA guidelines[55], ELISPOT assays were performed using validated, standard operating protocols. Vaccinated female mice were euthanized using isofluorane, and spleens were immediately processed into single cell suspensions by pressing through 100 μm screens in complete RPMI (RPMI + HEPES (Bibco: 22400–089) supplemented with L-glutamine, β-mercaptoethanol, penicillin and streptomycin, sodium pyruvate (Hyclone: SH30239.01) and 10% FBS). Red blood cells were lysed using ACK lysis buffer, and fresh splenocytes were counted on a Guava cytometer using Viacount (Cat#: 4000–0130). Splenocytes were diluted to 5 x 10^6^ or 10^7^ live cells/ml (usually between 75–85% of splenocytes were viable) in complete RPMI. ELISPOT plates (MSIP, Millipore:MSIPS4W10) were coated with 10 μg/ml of anti-mouse IFN-γ (mAb AN18, Mabtech: 3321-3-1000) or IL-2 (Mabtech: 3441-2H) capture antibody and incubated overnight at 4°C. Plates were washed and blocked for 2 hours at 37°C, 5% CO_2_ with complete RPMI. Within 2 hours of mice being sacrificed, 5 x 10^5^ to 10^6^ live, processed splenocytes were added to each well and stimulated with 0.002–20 μg/ml of the indicated peptides, media alone, 5 μg/ml Concanavalin A (Sigma: C5275) as positive control, or suspensions of 10^5^ tumor cells/well. Cultures were incubated for 20 hours at 37°C and 5% CO_2_. Plates were washed, 1 μg/ml of secondary antibody (biotinylated anti-IFN-γ: mAbR4-6A2, Mabtech: 3321-6-250, or biotinylated anti-IL-2:Mabtech: 3441-2H) was added, and plates were incubated for 2 hours at 37°C. Plates were developed using Vector Labs’ Vectastain ABC Elite kit (Cat#: PK-6100) and Vectastain AEC substrate reagent (Cat#: SK-4200) according to manufacturer’s protocol and scored with an automated plate reader (AID) using preset parameters that have been validated over several experiments.

### Intracellular cytokine staining (ICS) and cell sorting

Mice were vaccinated and euthanized as described above. Fresh splenocytes (2 x 10^6^ cells/1ml/well) were incubated with 20 μg/ml of cognate mutant peptide, or media alone, in 24 well plates. For ICS, splenocytes were incubated for 2 hours at 37°C and 5% CO_2_. GolgiPlug (BD: 51-2301KZ) was added, and the cells incubated for an additional 11 hours. Cells were then processed according to the manufacturer’s protocol using the FoxP3/Transcription Factor Staining Set (eBioscience, Cat#: 00–5523). Briefly, cells were harvested and stained for 30 minutes with fixable viability dye (0.5 μl/1ml sample) (eF780, eBioscience: 65-0865-18), washed and stained for 30 minutes with surface antibodies (CD4-V450 1/200, BD: 560468; CD8-PE 1/500, TONBO Biosciences: 50-0081-U100; MHCII-FITC 1/200, eBiosciences: 1250166), fixed and permeabilized, and stained for 30 minutes with IFN-γ antibodies (IFN-γ-APC 1/200, eBiosciences: 17–5743). Analytical flow cytometry was performed on a BD FACSCalibur flow cytometer and analyzed using FlowJo (vX.07). For cell sorting, cells were harvested from 24 well plates after 24 hours of stimulation with peptide. Cells were filtered through a 40μm nylon filter, stained with viability dye-eFluor780 (0.5 μl/1ml solution), washed, and stained with surface antibodies (CD4-V450 1/200; CD8-V500 1/50, BD: 560776; MHCII-FITC 1/200; 41BB-PE 1/100, BioLegend:107105; OX40-APC 1/100, BioLegend: 119409). Cells were sorted using a BD Influx cell sorter, and sorted cells were expanded in 100U/ml IL-2 (eBiosciences: 34–8021) in complete RPMI.

### Neoantigen prediction in the TCGA lung cancer and HGSC datasets

TCGA mutation annotation files were parsed, and HLA alleles were predicted from RNA-seq data for HGSC, lung adenocarcinoma (LUAD), and lung squamous cell carcinoma (LUSC) tumors as previously described[21]. SNVs were further filtered by requiring at least one read in the RNA-seq bam file to have the mutated base. Samtools v0.1.17 was used to extract reads covering the mutation site from the indexed bam files, and a custom python script checked for the mutated base. Epitope predictions were performed for all 8-11mer peptides relative to autologous *HLA-A* allele(s) using NetMHCpan v2.8[52]. An IC50 value of < 100 nM was used as the threshold for calling high affinity epitopes[18]. Since only *HLA-A* alleles were called due to challenges in calling unambiguous *HLA-B* and *C* alleles from 50bp sequencing data provided by TCGA, values were multiplied by 3 to account for 3 HLA loci (*HLA-A*, *B*, *C*). To estimate the likelihood of a patient with a given number of predicted neoantigens containing at least one authentic neoantigen, we applied the findings of a study showing that only 8% of peptides that bind MHCI with IC50 < 100 nM proved to be authentic (ie. 92% were not authentic)[18]. We calculated the chance of a patient containing 0 immunogenic mutations using the equation 0.92^N^, where “N” equals the number of predicted neoantigens. Subtracting from one, we arrived at the percent likelihood of a patient with N predicted neoantigens containing at least one authentic neoantigen.

## Results

### Identification and validation of tumor-specific mutations in the ID8-G7 tumor line

With the goal of studying an ovarian cancer model with a neoantigen repertoire similar to human tumors, we investigated a variant of the ID8 tumor line (ID8-G7). Because the ID8 line was generated by *in vitro* serial passage, it was not expected to harbor the high number of mutations found in carcinogen-induced tumors. However, since the transformation process occurred entirely *in vitro* and in the absence of any immunological pressure, we reasoned it might have given rise to neoantigens with unnaturally high MHC affinities. In contrast, the ID8-G7 line underwent one round of *in vivo* passage in a syngeneic (C57Bl/6), immunocompetent mouse[47], which should have resulted in a repertoire of neoantigens that more closely resembles tumors from immunocompetent ovarian cancer patients. While this might have eliminated expression of some immunogenic, dispensable mutations (i.e., passenger mutations), any essential driver mutations should have been retained. To provide a positive control antigen, the ID8-G7 line expresses the CD8 T cell epitope SIINFEKL as a chimeric construct with the rat *neu* protein (Neu^OT-I/OT-II^)[47]. As hosts, we used *MMTV/neu*^*OT-I/OT-II*^ transgenic mice, which are tolerant to SIINFEKL[54]. After intraperitoneal injection into *MMTV/neu*^*OT-I/OT-II*^ mice, the ID8-G7 tumor line gives rise to aggressive, widely disseminated cancer, characterized by extensive ascites[47]. However, near-complete regression of advanced disease can be achieved by adoptive transfer of SIINFEKL-specific CD8 T cells (OT-I T cells), demonstrating the sensitivity of ID8-G7 tumors to immunological control[47]. Thus, the ID8-G7 model provided a well-controlled system for evaluating neoantigens as targets for immunotherapy.

**Fig 1:**
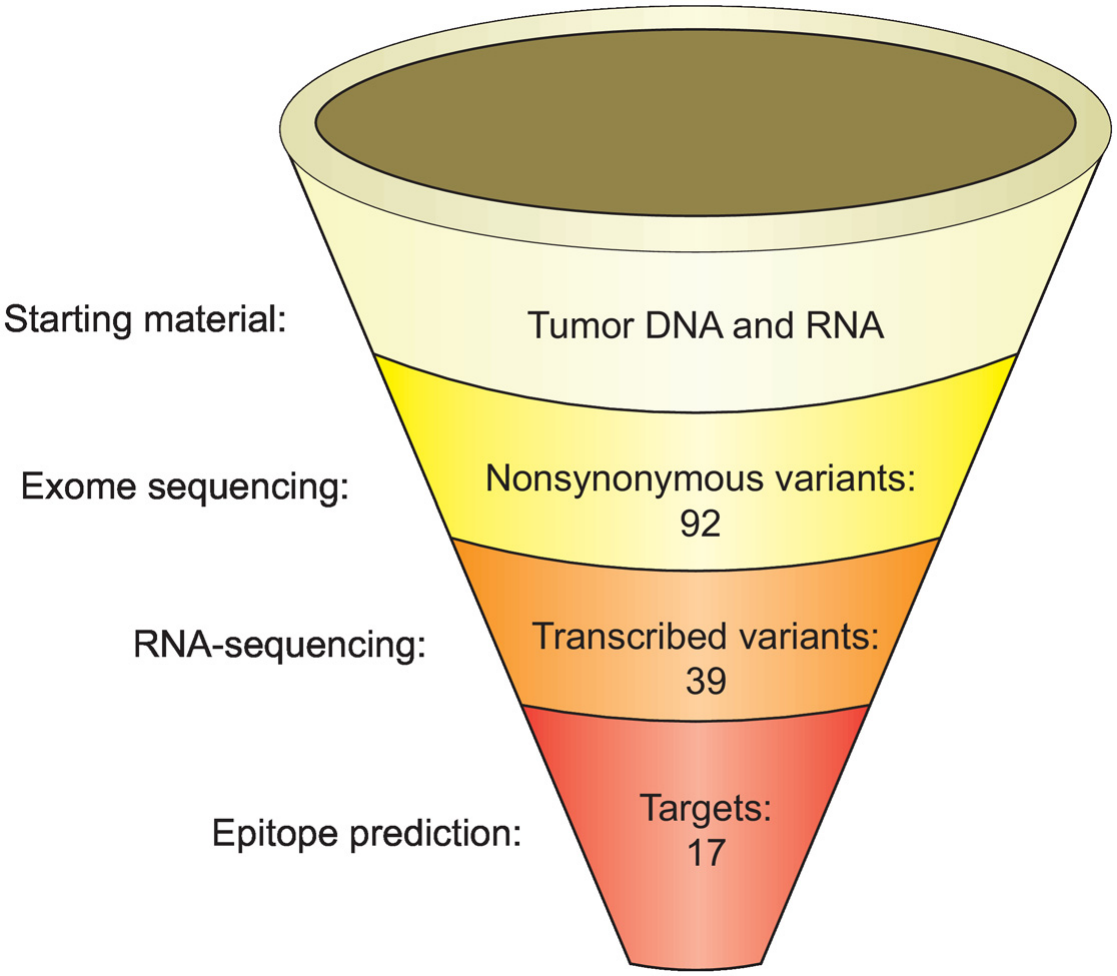
Experimental pipeline for identifying and predicting immunogenic mutations in the ID8-G7 tumor line. Mutations were identified by whole exome sequencing, and expression was assessed using RNA-seq. Epitope prediction was performed using NetMHCpan2.4[52]. Starting with 92 mutations in genomic DNA, 17 mutations within peptides predicted to bind MHCI (IC50 < 1500 nM) were advanced to subsequent experiments.

Exome sequencing was performed on genomic DNA from both the ID8-G7 tumor cell line and C57Bl/6 murine splenocytes. In addition, RNA-seq was performed on the ID8-G7 tumor line. Exome sequencing generated 127 million reads that mapped to the reference mouse genome, resulting in an average of 89-fold coverage. Paired-end RNA-seq generated 158 million mapped reads (sequencing bam files are available at the NCBI sequence read archive with the following accession number: SRP069102). After removing variants present in either the control mouse genome or dbSNP138, we identified 92 non-synonymous tumor-specific coding variants (Fig 1). Of these, 42 variants were present in at least 1 read within the RNA-seq data, indicating that they were transcribed (S1 Table). We excluded non-sense mutations, as these were not expected to give rise to T cell epitopes. This left 39 non-synonymous, transcribed, missense or insertion/deletion (Indel) variants (Fig 1). The amino acid sequences encompassing the mutations were queried using epitope prediction software (NetMHCpan2.4[52]). Using a relaxed cutoff of IC50 < 1500 nM, we identified 17 mutations predicted to give rise to at least one MHCI binding epitope, and these were advanced to vaccination experiments (Table 1). One additional predicted MHCI binding mutation (in the gene 1110021LRik) could not be assessed due to difficulties synthesizing the corresponding peptide. All of the targeted mutations qualified as intermediate affinity binders, as their predicted IC50 scores ranged from 103–1160 nM (Table 1).

**Table 1:** Predicted epitopes and 29mer peptides selected for vaccination study. Each mutation that was found in a peptide predicted to bind to H-2Kb or H-2Db with an IC50 < 1500 nM was annotated. Shown for each mutation are the amino acid substitution, predicted highest binding epitope, binding score, binding H-2 allele, the RNA-seq read count supporting the mutated and wild type allele, and the 29mer peptide used for vaccination.

Gene	aa substution	Predicted epitope	Score	H-2	¶Reads WT	¶Reads Mut	29mer peptide
1110021L09Rik	Y284S	LSLS**S**AISSPL	668	Kb	38	8	TGYVGLVFLGLSLS**S**AISSPLFGLLSDKM*
B4galt3	A21P	**P**VMMYLSLGGF	738	Kb	53	10	ERPCTLALLVGSQL**P**VMMYLSLGGFRSLS
Bat1a	S4P	MSF**P**GFFVV	699	Kb	79	15	MSF**P**GFFVVPSPSSSVGN**
Celsr1	P1063A	LNFTGAQV**A**RF	1100	Kb	33	33	VDIFDKLNFTGAQV**A**RFEDIQEELPRELE
Cul2	E222G	EYYKQ**G**ASNLL	1160	Kb	63	120	VSPFLTETGEYYKQ**G**ASNLLQESNCSQYM
D6Ertd527e	P233L	SS**L**RPSNRGSI	609	Db	2	1	SNTTTSSNSQSNSS**L**RPSNRGSISNYSNS
Dync1h1	V3088E	TSPALFNRC**E**L	434	Kb	208	114	KDRAATSPALFNRC**E**LNWFGDWSTEALYQ
Gm608	P947S	S**S**IESQSL	674	Kb	0	18	DMKSCTSANVLTPS**S**IESQSLVSQVSGLS
Ipo13	A165S	**S**LLELLTVL	1033	Db	1	176	QAEDSPVDSQGRCL**S**LLELLTVLPEEFQT
Mtdh	532_535del	**VTRHRQAQV**	614	Kb	0	60	SITLSKGDSDNSSSHVTRHRQAQVKC***
Mterfd1	K184T	I**T**QILLFL	1060	Kb	211	56	AANLLLRLDFEKHI**T**QILLFLKDLGLEDN
Myo9a	M2394V	VIVRLPS**V**	187	Kb	14	9	YPSPSSPVIVRLPS**V**SDVPEETLSSETAM
Pkp4	T1012I	FI**I**PVSTL	1119	Kb	212	93	SIYKKDGWNQNHFI**I**PVSTLERDRFKSHP
Pla2g4a	M234I	STWY**I**STL	352	Kb	204	88	CATYIAGLSGSTWY**I**STLYSHPDFPEKGP
Plcxd2	G280A	**A**GLKNTLV	634	Db	0	9	ILTPRVKTIARGLV**A**GLKNTLVHRNLPAI
Ptgfr	C121W	ISMVFSGL**W**PL	103	Kb	9	18	LCSIFGISMVFSGL**W**PLFLGSAMAIERCI
Rpl5	V159F	TGNK**F**FGAL	499	Kb	687	1753	YLDAGLARTTTGNK**F**FGALKGAVDGGLSI
Tle1	H393Y	AAYAGL**Y**SM	179	Db	0	39	NGELTSPGAAYAGL**Y**SMSPQMSAAAAAAA
OVA	N/A	**SIINFEKL**	392	Kb	N/A	N/A	PDEVSGLEQLESIINFEKLTEWTSSNVME****

^¶^ Number of reads in RNA-seq data with the wild type (WT) or mutant (Mut) nucleotide

* Peptide not tested due to inability to synthesize

** Mutation occurred in the 4th residue so 18mer peptide was assessed to ensure coverage of all possible mutated 15mers

*** Frameshift deletion

**** Model antigen used as positive control

### Induction of mutation-reactive T cells by peptide vaccination

**Fig 2:**
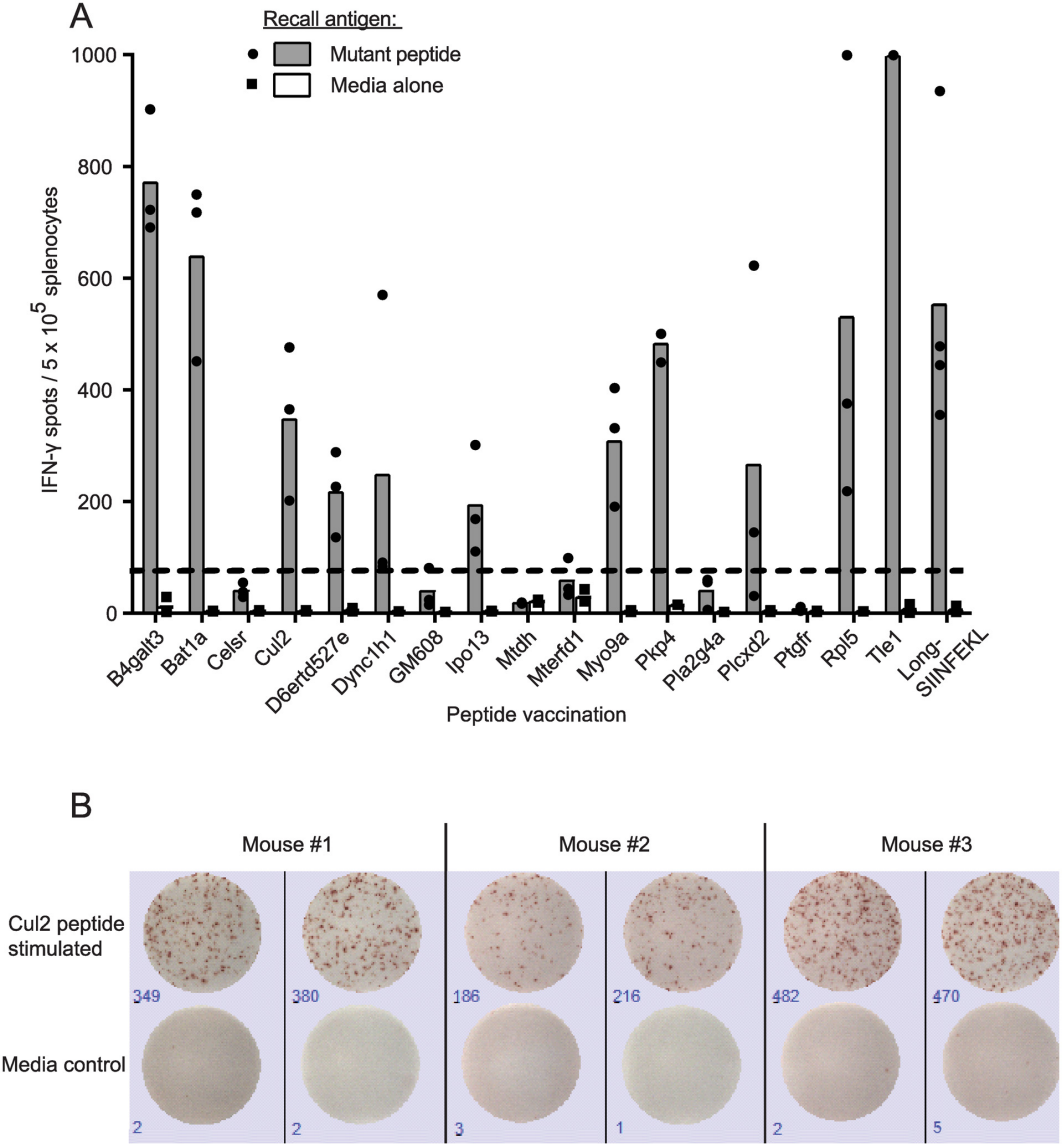
Immunogenicity of mutant peptides. Mice (n = 3 per group) were vaccinated daily on days 0–3 and 21–24 with mutant 29mer peptides (50 μg, one peptide per group) and poly(I:C) (10 μg). On day 28, mice were euthanized, and splenocytes (10^6^ cells/well) were stimulated in duplicate with mutant 29mer peptides (20 μg/ml) or media, and assessed by IFN-γ ELISPOT. Positive control mice were vaccinated with a 29mer peptide (long-SIINFEKL) encompassing the known OVA_257-264_ epitope. **A.** Eleven of 17 mutant peptides elicited robust T cell responses. Dots represent responses in individual mice, and bars represent the mean for three mice. The dashed line shows the threshold for positivity (3 x maximum background for splenocytes in media alone determined post-hoc). **B.** Representative ELISPOT wells from 3 mice vaccinated with mutant Cul2 peptide and stimulated in duplicate with mutant Cul2 peptide or media alone.

**Fig 3:**
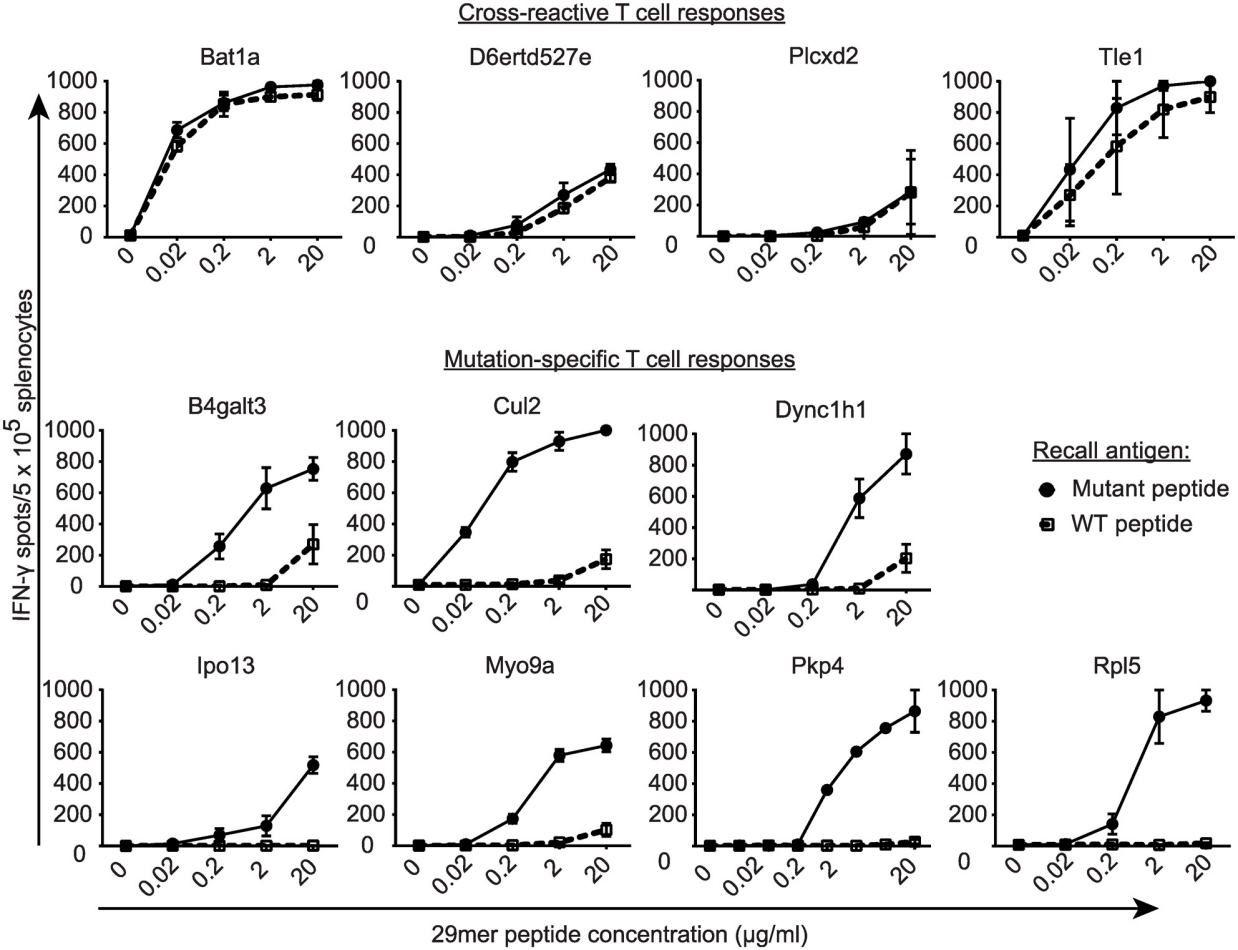
T cell specificity for mutant versus wild type peptides. Mice (n = 2 per group) were vaccinated as in Fig 2. On day 28, mice were euthanized, and splenocytes (5 x 10^5^ cells/well) were stimulated in duplicate with titrated concentrations of mutant or wild type 29mer peptides in IFN-γ ELISPOT wells. Dots and bars represent the mean and range of responses, respectively.

**Fig 4:**
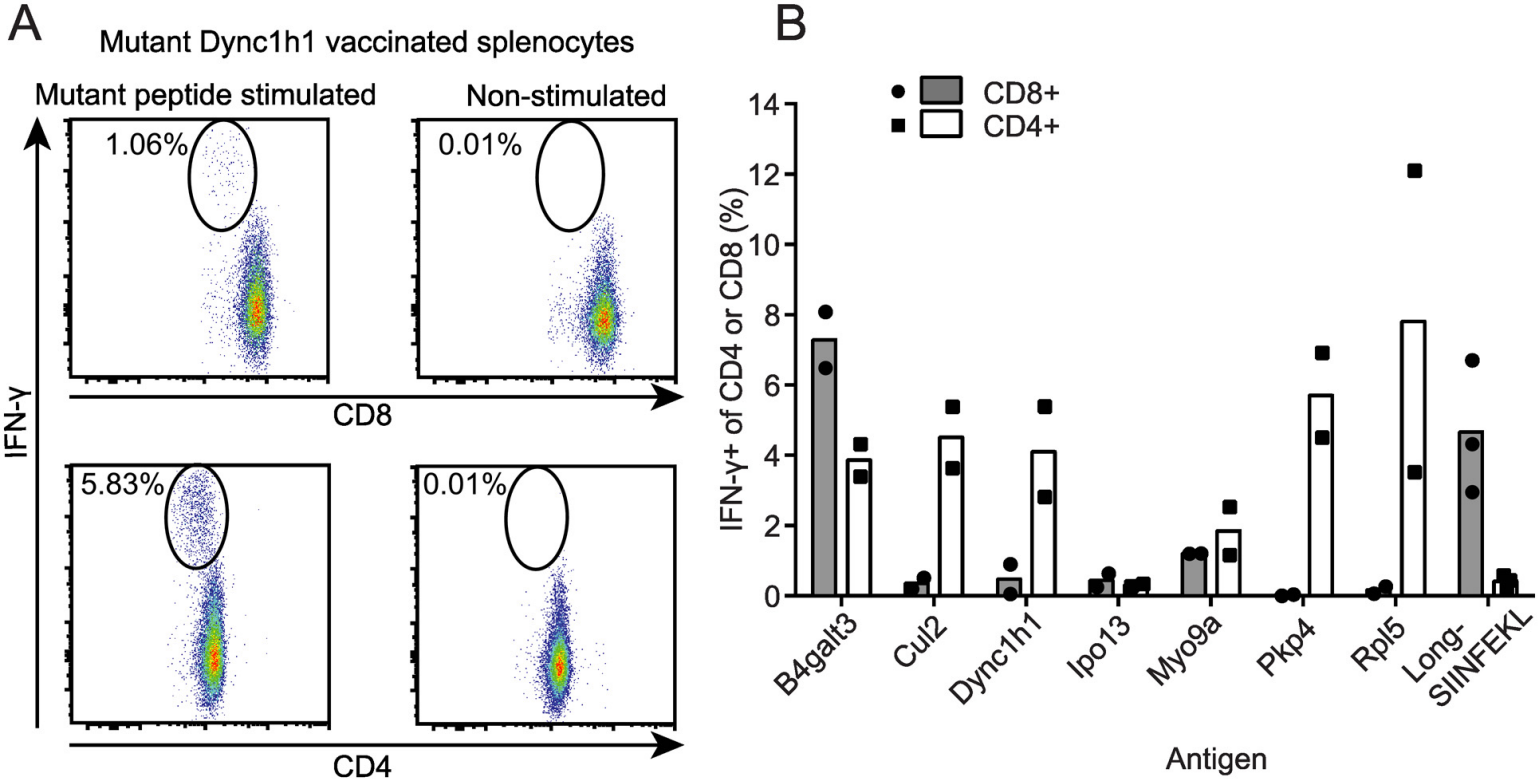
Determination of CD4 versus CD8 T cell responses to mutant peptide vaccines. Mice (n = 2 per group) were vaccinated as in Fig 2. On day 28, splenocytes were harvested from vaccinated mice, incubated overnight in media containing the indicated mutant peptides or media alone, and analyzed by flow cytometry. Cells were gated on viability and lack of MHCII expression (activated murine T cells are MHCII negative). The percentage of IFN-γ-secreting CD8 and CD4 T cells was determined. **A.** Example of a mixed T cell response (CD8 *top* and CD4 *bottom*) to mutant Dync1h1 29mer peptide versus media alone. **B.** Summary of data for 7 mutant peptides and the positive control peptide long-SIINFEKL. Bars and dots represent mean and individual responses, respectively.

To determine whether any of the 17 predicted neoantigens were immunogenic, naive C57Bl/6 mice were vaccinated with 29mer peptides with the mutated residue in the central position. By using 29mer peptides, intracellular antigen processing could potentially produce any mutation-bearing epitope of 15 amino acids or less. Each group of mice was vaccinated with one of the 17 mutant peptides and 10 μg poly(I:C) on four consecutive days as previously described[56], followed by four additional daily inoculations three weeks later. T cell responses were assessed by IFN-γ ELISPOT. This vaccination schedule elicited robust T cell responses to 29mer peptides encoding SIINFEKL, as well as mutant Spas1, a well characterized tumor neoantigen from the TRAMP-C2 tumor model[57] (S1 Fig). When used to target ID8-G7 tumor-specific mutations, vaccination elicited T cell responses to 11/17 mutant peptides (Fig 2). For these 11 mutant peptides, cross-reactivity to the wild type (WT) version of the peptide was assessed by titrating the WT and mutant peptides in IFN-γ ELISPOT assays. For 4/11 mutations, T cells responded to the mutant and WT peptides at similar concentrations, indicating a high degree of cross-reactivity (Fig 3). For the remaining 7/11 mutations, T cells showed a > 100-fold higher sensitivity to the mutant peptide compared to the WT peptide. Intracellular cytokine staining revealed that 5/7 of these responses involved both CD4 and CD8 T cells (Fig 4), with CD4 T cells dominating the responses in 4/5 cases. The remaining 2/7 responses were mediated by CD4 T cells alone. Thus, 7/17 (41%) of mutant peptides elicited a mutation-specific T cell response after vaccination, and most responses were dominated by CD4 T cells. These seven mutations were advanced to further studies.

### Prophylactic and therapeutic vaccinations of tumor-bearing mice

**Fig 5:**
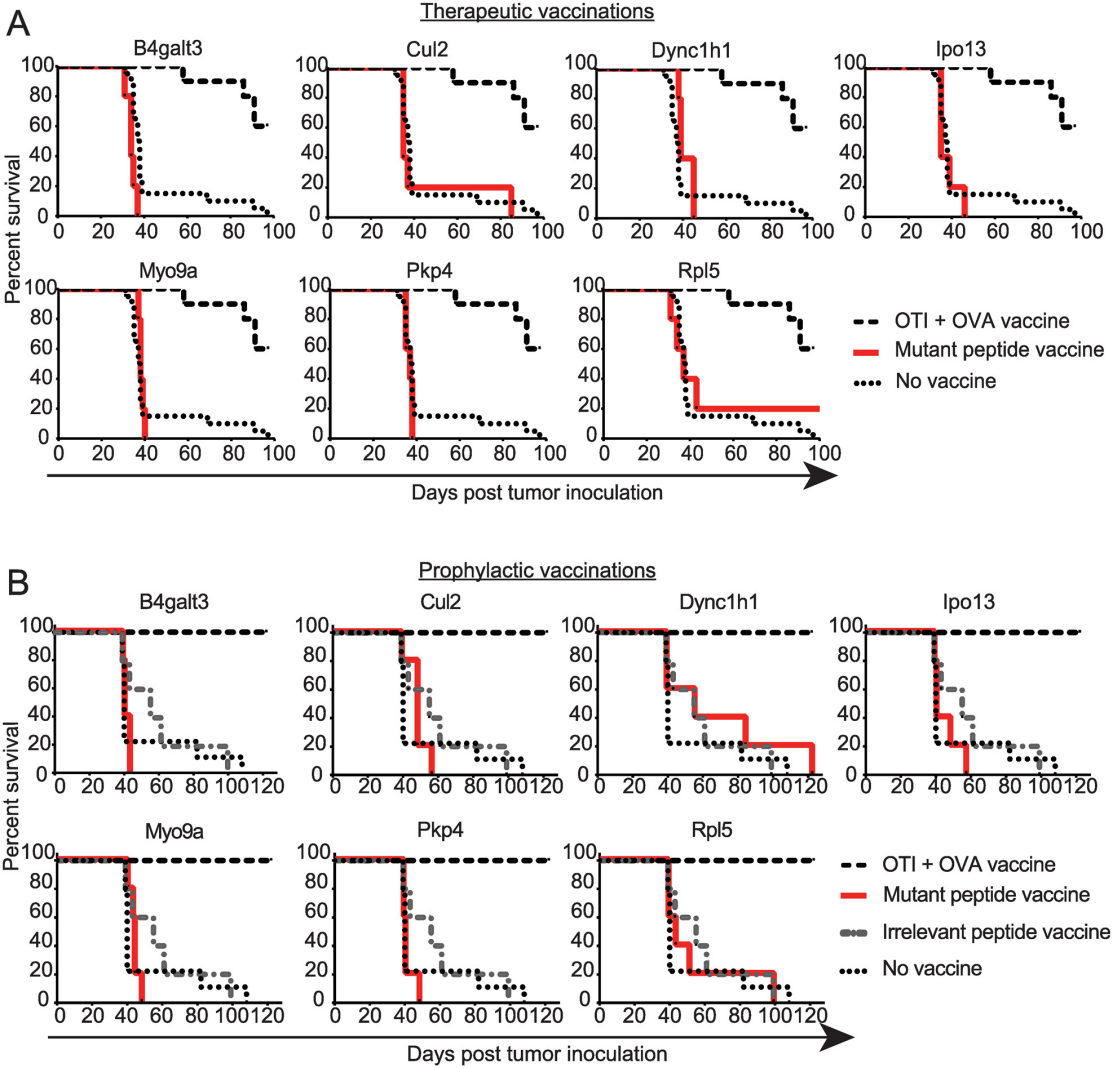
Therapeutic and prophylactic vaccination with mutant peptides. **A.** For therapeutic vaccination, mice (n = 5 per group) were inoculated with ID8-G7 tumor cells (10^6^ cells/mouse) on day 0 and vaccinated on days 3–6 with individual mutant peptides (50μg) and poly(I:C) (10μg). As a positive control, one group of mice received adoptive transfer of OT-I splenocytes on day 2, followed by vaccination on days 3–6 with OVA protein (100 μg) and poly(I:C) (10 μg). Non-vaccinated mice served as negative controls. Each graph represents one group of 5 mice vaccinated with a single mutant 29mer peptide. The same group of positive and negative control mice were used for each graph. Mice were euthanized once they displayed abdominal distension due to ascites. **B.** For prophylactic vaccination, mice (n = 5 per group) were vaccinated on days -28 to -25 and -7 to -4 with mutant 29mer peptides (25 μg, 1 peptide per group) and poly(I:C) (10 μg). As a positive control, one group of mice received adoptive transfer of OT-I splenocytes on day -8 followed by vaccination on days -7 to -4 with OVA protein (100 μg) and poly(I:C) (10 μg). As negative controls, one group of mice was vaccinated with an irrelevant peptide, and a second group of mice received no vaccination. On day 0, mice received intraperitoneal inoculation of 10^6^ ID8-G7 tumor cells. Mice were euthanized once abdominal distension due to ascites was observed.

To test whether vaccine-activated mutation-specific T cells could induce regression of ID8-G7 tumors, mice bearing three day old tumors were vaccinated on four consecutive days with individual mutant peptides and poly(I:C). As expected, 9/10 positive control mice that underwent ACT with OT-I followed by vaccination with OVA and poly(I:C) remained tumor free 80 days post tumor implantation (Fig 5a). In contrast, none of the seven mutant peptide vaccines increased survival of mice beyond that seen with non-vaccinated control mice (Fig 5a). We performed similar vaccination experiments with peptides that were minimally-immunogenic or elicited cross-reactive T cells, and these eight vaccines also failed to increase survival (S2 Fig).

Recognizing that cancer vaccines are not always effective for treating established tumors, we also tested mutant peptides in a prophylactic vaccination setting. Mice were vaccinated with mutant peptides on days -28 to -25 and -7 to -4, followed by intraperitoneal injection of ID8-G7 tumor cells on day 0. One animal per group was euthanized on day 0, and T cell activation was confirmed in all cases by IFN-γ ELISPOT. Mice that received adoptive transfer of OT-I and vaccination with OVA remained tumor free for > 300 days, confirming that ID8-G7 tumor cells were susceptible to T cell-mediated rejection. However, none of the mice vaccinated with mutant peptides showed a significant difference in survival compared to mice that received an irrelevant peptide or no vaccination (Fig 5b). Thus, despite eliciting robust T cell responses, none of the mutant peptide vaccines demonstrated a significant anti-tumor effect in either the prophylactic or therapeutic settings.

### In vitro tumor recognition experiments

**Fig 6:**
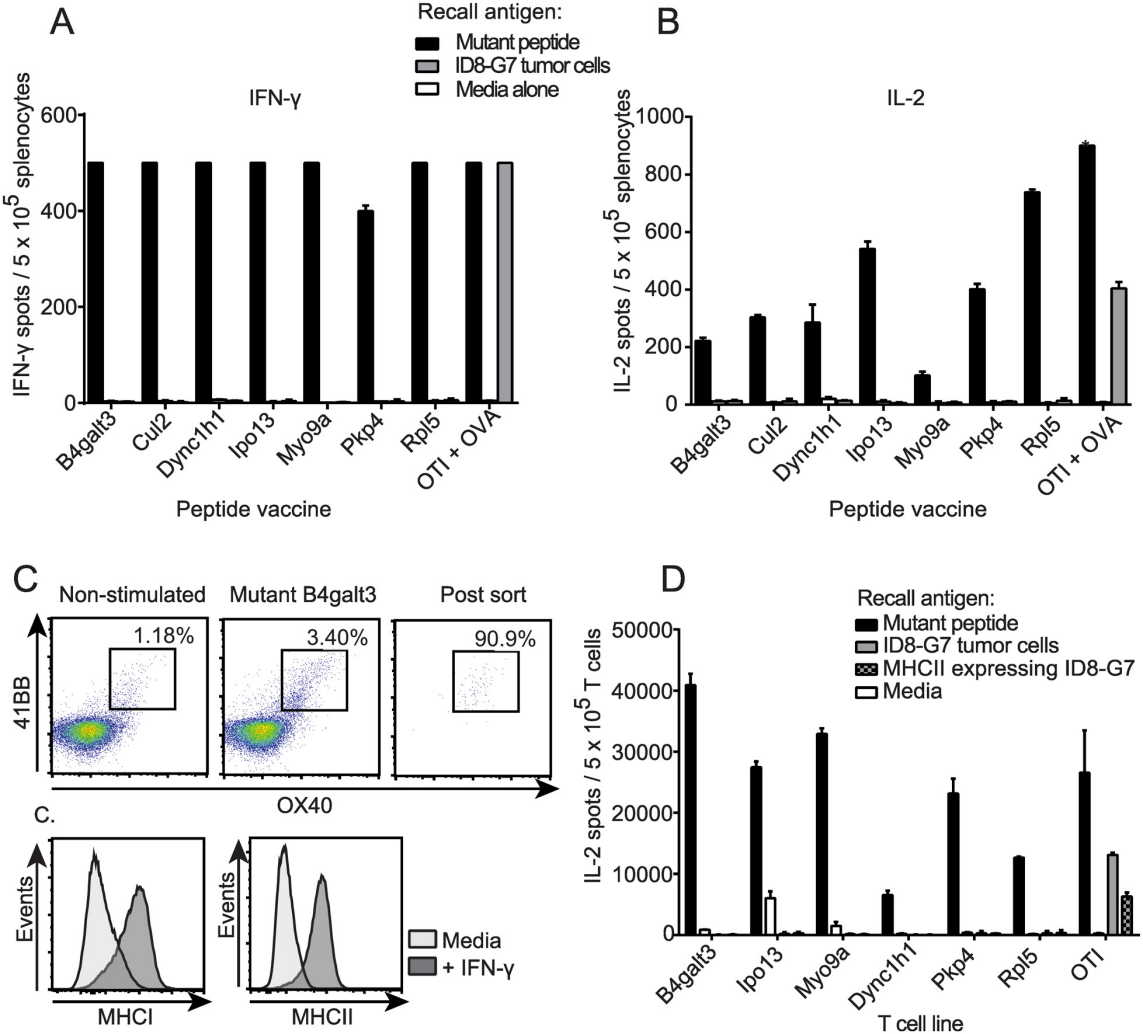
*In vitro* assessment of mutation-reactive T cells for recognition of ID8-G7 tumor cells. Mice were vaccinated as in Fig 2, and splenocytes were harvested on day 28. OT-I splenocytes were used as positive controls. Splenocytes (5 x 10^5^ per well) were stimulated in duplicate with cognate mutant peptide (20 μg/ml), media alone, or ID8-G7 tumor cells (10^5^ cells per well) and assessed by **A.** IFN-γ ELISPOT, or **B.** IL-2 ELISPOT. **C.** To assess tumor recognition by mutation-reactive CD4 T cells, 4-1BB^+^OX40^+^ CD4 T cells from the above mice were FACS purified and cultured in recombinant murine IL-2 (100 U/ml). ID8-G7 tumor cells were incubated in IFN-γ (100 U/ml) for 72 hours to cause upregulation of MHCI and MHCII. **D.** Sorted and expanded CD4 T cells (10^4^ per well) were stimulated in duplicate with either 20 μg/ml of cognate mutant peptide, media, ID8-G7 tumor cells or ID8-G7 tumor cells incubated in IFN-γ for 72 hours. OT-I T cells were used as a positive control for tumor cell recognition. Bars and lines represent mean and standard deviation of duplicate wells, respectively.

To investigate why mutant peptide vaccines failed to provide tumor control *in vivo*, we assessed whether vaccine-induced T cells recognized ID8-G7 tumor cells *in vitro*. As before, mice were vaccinated with mutant peptides, and splenocytes were harvested on day 28. In IFN-γ (Fig 6a) and IL-2 (Fig 6b) ELISPOT assays, splenocytes from immunized mice responded strongly to corresponding mutant 29mer peptides but showed no response to ID8-G7 tumor cells. As a positive control, SIINFEKL-specific T cells responded to both peptide and ID8-G7 tumor cells (Fig 6a and 6b). To further assess CD4 T cell responses, splenocytes from vaccinated mice were stimulated *in vitro* with cognate peptides, sorted based on upregulation of 41BB and OX40 (Fig 6c), and expanded with IL-2. ID8-G7 tumor cells were pre-treated with IFN-γ, which resulted in upregulation of MHCII (Fig 6c). Tumor cells and T cell lines were co-incubated overnight and assessed by IL-2 ELISPOT assay. None of the mutation-reactive CD4 T cells demonstrated recognition of ID8-G7 cells (Fig 6d). Collectively, the results of these tumor recognition experiments suggest that none of the assessed mutations gave rise to epitopes that were naturally presented by MHCI or MHCII on ID8-G7 tumor cells in sufficient quantity to stimulate T cells.

### Comparison of predicted immunogenic mutations in human HGSC versus lung cancer

**Fig 7:**
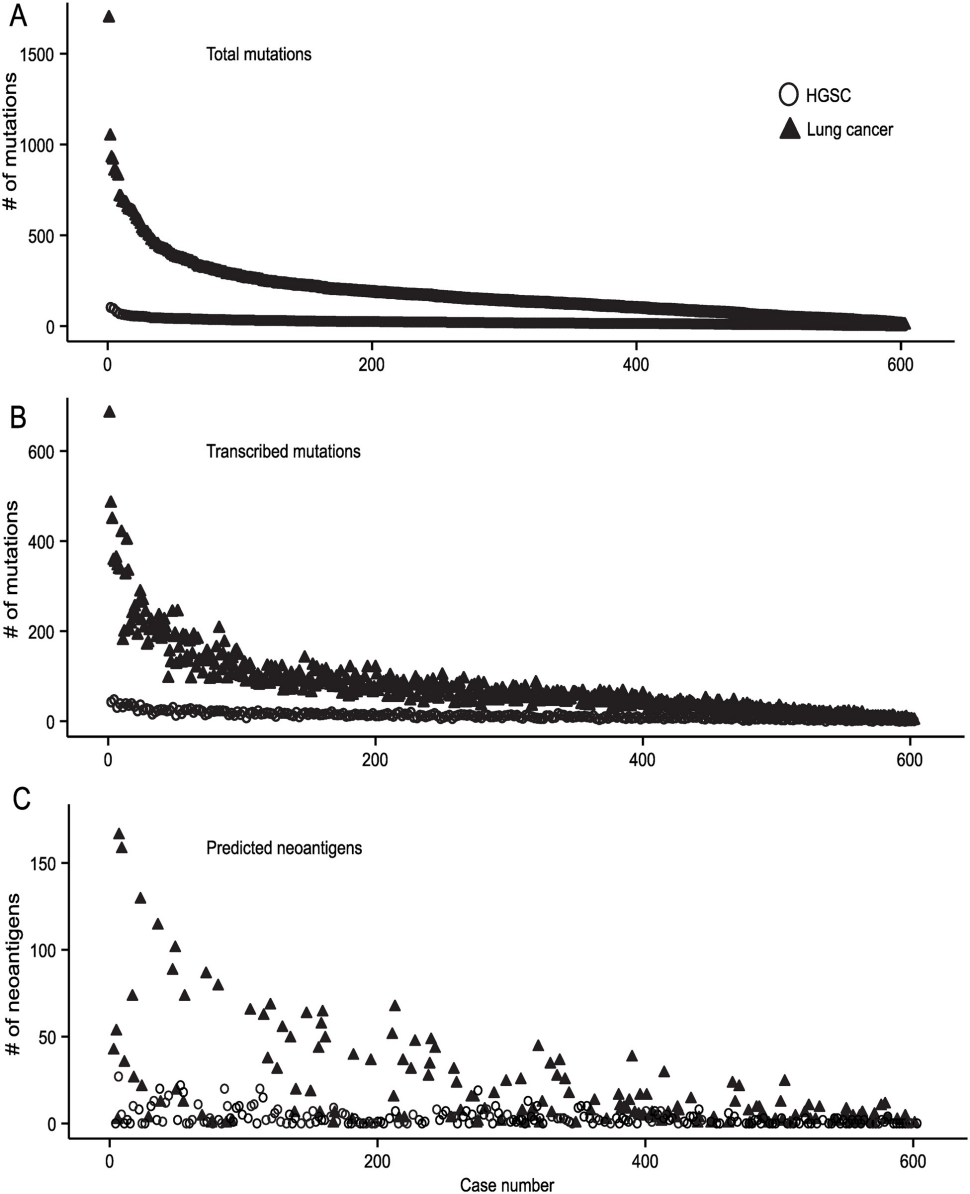
Mutation and neoantigen loads in the human HGSC and lung cancer TCGA datasets. Individual cases are aligned on the X axes in rank order according to the total number of mutations identified by whole exome sequencing (panel A); the same order of cases was used in panels B and C. Y axes indicate the number of mutations meeting the indicated criteria for each panel. Lung cancer cases (n = 603) are shown as solid black triangles, whereas HGSC cases (n = 274) are shown as hollow circles. **A.** Somatic, non-synonymous mutations identified by whole exome sequencing (total mutations). **B.** Somatic, non-synonymous mutations with at least one corresponding read in the RNA-seq data (transcribed mutations). **C.** Peptides predicted to bind autologous *HLA-A* with an IC50 < 100nM and containing somatic, non-synonymous, transcribed mutations. Only samples with unambiguous *HLA-A* allele calls are shown (lung n = 158, HGSC n = 220).

Our findings in the ID8-G7 tumor model suggested that mutations with intermediate affinity for MHCI (IC50 scores from 103–1160 nM) have a low probability of giving rise to authentic, therapeutically relevant epitopes. Instead, high affinity epitopes may be required, as supported by numerous murine and human studies[58–62]. To explore the implications of this for human HGSC, we interrogated the TCGA HGSC dataset (TCGA-OV) to determine the proportion of tumors with somatic point mutations predicted to give rise to high affinity MHCI binding peptides (IC50 < 100 nM). For comparison, we assessed the lung carcinoma datasets as examples of tumor types with high mutation burdens. Candidate neoantigens were identified based on the following criteria: 1) the mutation being present in the tumor but not in matched normal tissue (Fig 7a); 2) at least one read from RNA-seq data supporting expression of the mutated allele (Fig 7b); and 3) a predicted MHCI binding score of < 100 nM for at least one of the autologous *HLA-A* alleles (Fig 7c). Only samples with unambiguous *HLA-A* allele calls were analyzed for immunogenicity due to challenges in calling unambiguous *HLA-B* and -*C* alleles from 50bp sequencing data. Since only *HLA-A* alleles were assessed, we multiplied our results by three to account for the three MHCI loci in humans (*HLA-A*, -*B*, and–*C*). Using these parameters, the median number of predicted MHCI neoantigens was 6 for HGSC compared to 27 for lung cancer. Empirical studies of viral epitopes indicate that only 8% of peptides that bind MHCI with IC50 < 100 nM prove to be authentic epitopes (i.e., are naturally processed and presented by MHCI)[18]. Therefore, after multiplying by 0.08, we estimated that the expected number of authentic MHCI neoantigens in a tumor with median number of predicted epitopes was 0.48 for HGSC compared to 2.16 for lung cancer. Based on these assumptions, a patient with the median number of mutations would have only a 39% chance of containing one or more authentic MHCI-binding neoantigens in HGSC compared to an 89% chance in lung cancer. Put another way, 12% of HGSC cases versus 51% of lung cancers would have ≥ 90% likelihood of containing at least 1 neoantigen. Thus, results from both the ID8-G7 tumor model and TCGA data reveal the potential limitations of targeting neoantigens in HGSC.

## Discussion

We investigated the concept of neoantigen-specific vaccination for ovarian cancer using a murine ovarian tumor model and *in silico* analysis of human HGSC data from TCGA. We subjected the ID8-G7 tumor line to whole exome sequencing, RNA-seq, and epitope prediction and found 92 mutations, 39 of which resulted in amino acid substitutions and were transcribed. By *in silico* analysis, none of the transcribed mutations gave rise to neoepitopes predicted to bind MHCI with high affinity (IC50 < 100nM); therefore, mice were vaccinated with mutant peptides containing MHCI epitopes with intermediate predicted affinity (IC50 scores of 103–1160 nM). Although 7/17 peptide vaccines elicited robust, mutation-specific CD4 and CD8 T cell responses, the mutation-reactive T cells failed to recognize ID8-G7 tumor cells *in vitro*. Moreover, neither prophylactic nor therapeutic vaccination resulted in effective anti-tumor activity *in vivo*. When combined with published results[32,33,59], our findings indicate that high affinity, MHCI binding epitopes (IC50 < 100 nM) may be required to elicit neoantigen-reactive CD8 T cell responses capable of mediating tumor regression. Yet our bioinformatic analysis of TCGA data revealed that only 12% of HGSC cases had ≥ 90% likelihood of harboring one or more neoantigen. Thus, our study highlights a major challenge associated with neoantigen-specific vaccines in HGSC and potentially other malignancies with intermediate to low mutation loads.

Although we used an established approach to predict CD8 T cell neoantigens, it is possible that relevant MHCI-binding neoantigens may have been overlooked. For example, using an MCA-induced mouse fibrosarcoma model, one group reported that therapeutically relevant neoantigens did not necessarily have the highest predicted MHCI binding affinity but rather the greatest difference of MHCI binding affinity between the mutant and wild type peptide[63]. However, two studies have shown that the majority of published human CD8 T cell neoantigens identified by non-biased cDNA library screens could be predicted based on the binding affinity of the mutant peptide alone[61,62]. In one of these studies, 22/31 (71%) of validated neoepitopes were predicted to bind autologous HLA with IC50 <100 nM, while most of the remaining neoepitopes (5/31, 16%) had predicted IC50 scores between 100–500 nM[61]. Furthermore, authentic CD8 T cell neoantigens identified in all other NGS-guided preclinical studies could be identified using high predicted binding scores (IC50 ≤ 100 nM)[32,33,64]. Thus, since we evaluated every predicted epitope with an IC50 score < 1500 nM, it seems unlikely that authentic MHCI-binding neoantigens were missed by our analysis.

Recently, several groups have highlighted the importance of CD4 T cell responses to neoantigens. In one study, a neoantigen-targeted vaccine induced activation of CD4 T cells that mediated rejection of B16F10 melanoma[31]. Moreover, neoantigen-specific CD4 T cells are commonly found among tumor-infiltrating lymphocytes in melanoma patients[24]. Furthermore, adoptive transfer of a near-clonal population of neoantigen-reactive CD4 T cells resulted in regression of a metastatic cholangiocarcinoma[7]. Our neoantigen-specific vaccines were designed to allow processing of MHCII epitopes and activation of CD4 T cells, since all possible mutant epitopes up to 15 residues in length could be processed from a mutant 29mer peptide. Indeed, our data show that CD4 T cells were the dominant component in 6/7 mutation-specific T cell responses. However, none of these responses translated to anti-tumor efficacy *in vivo*. It is possible that we missed therapeutically relevant CD4 T cell neoantigens that were > 15 residues in length or present among the 22 expressed mutations not assessed in vaccination experiments. Unfortunately, MHCII epitope prediction algorithms are not yet sufficiently accurate for efficient vaccine design[65–67]; however, the need for such tools is becoming increasingly evident for cancer immunotherapy.

Since the ID8-G7 tumor line had been passaged in an immunocompetent mouse, highly immunogenic neoantigens present in the parental ID8 line may have been lost or silenced due to immune editing[68]. For example, immune editing of neoantigens was demonstrated in an MCA-induced sarcoma model, where a tumor that arose in an immune deficient mouse was found to carry a mutation which was lost upon transplantation of the tumor into an immunocompetent mouse[64]. Indeed, the ID8-G7 tumor genome contained four mutant genes with predicted mutant epitopes IC50 < 100 nM that were not transcribed (S2 Table). However, given the higher tumorigenic capacity of the ID8-G7 line compared to the parental ID8 line[47], it appears that expression of these mutations is not required for tumor cell proliferation, viability or metastatic spread. Thus, at best, they may represent passenger mutations with questionable value as therapeutic targets. Indeed, the importance of so-called “trunk” mutations rather than “branch” mutations in therapeutic responses to PD-1 blockade has been highlighted in a recent study in human lung cancer[69]. Given these theoretical considerations, one must concede that murine tumor models such as ID8-G7 inevitably have caveats with respect to immune editing. Ultimately, estimates of neoantigen load are best made in human cancers, where tumors develop under natural physiological conditions that include immune editing.

To this end, we used TCGA data to estimate the prevalence of neoantigens in HGSC and, as a comparator, lung cancer. We calculated that a tumor with the median number of predicted neoantigens would likely contain 2.16 authentic neoantigens in lung cancer versus 0.48 in HGSC, meaning that many HGSC tumors may be devoid of authentic neoantigens. This is consistent with our prior study, in which only 1 of 3 HGSC patients demonstrated a neoantigen-specific CD8 TIL response[12]. However, in addition to considering median values, it is important to recognize that mutation burden can vary by several orders of magnitude in some tumor types[19]. An extreme example of this phenomenon is endometrial cancer (EC), where patients with mutations in *POLE*, encoding a DNA proofreading enzyme, have 105-fold more predicted neoantigens than POLE non-mutated EC[70]. Another example is colorectal cancer, where cases with microsatellite instability (MSI) harbor dramatically more mutations than microsatellite stable tumors[71]. Other histological subtypes of epithelial ovarian cancer, in particular the clear cell and endometrioid subtypes, also exhibit MSI in a substantial proportion of cases[72]. However, HGSC exhibits a relatively narrow range of mutation burdens and lacks a hypermutated subtype[73]. Indeed, only 12% of HGSC cases have >90% chance of harboring at least one authentic neoantigen compared to 51% of lung cancers (Fig 7).

TIL are strongly associated with increased survival in HGSC[35], yet our studies (here and [12]) show that relatively few patients are likely to harbor neoantigens. If so, what antigens do HGSC TIL recognize? Several groups have demonstrated that TIL can recognize cancer testes[39], overexpressed[37], and differentiation antigens[40] in HGSC. Moreover, all studies to date that have used NGS to identify neoantigens (including the present study) have evaluated only point mutations and small indels. This may be an important technical limitation, as approximately half of HGSC harbor homologous recombination defects that can lead to high frequencies of indels, many of which may be too large for detection by convention WES[34,74]. Furthermore, HGSC is thought to be primarily driven by large genomic rearrangements and transcriptome aberrations [75,76], which can give rise to other classes of neoantigen involving gene fusions[77], intron retentions[78], and splice variants[79]. Such alterations can be highly immunogenic, as they deviate even further from the germ line sequence compared to point mutations. Typically, these types of variants are identified using RNA-seq, which at present is beset with high rates of false positive variant calls. Further improvement to RNA-seq library preparation and bioinformatic analysis will create access to new classes of neoantigens for immunotherapeutic targeting in HGSC and other chromosomally unstable cancers. Thus, while the “point mutanome” of HGSC may contain relatively few targets for immunotherapy, other classes of neoantigen await investigation. The strong prognostic significance of tumor-infiltrating lymphocytes in HGSC provides both impetus and optimism for these efforts.

## Supporting Information

S1 Fig T cell responses to peptide vaccination. **A.** Mice were vaccinated with 29mer peptides (50 μg) encompassing the SIINFEKL epitope and poly(I:C) (10 μg) on days 0–3 and 21–24, blood was harvested every 2–4 days, and blood draws were processed for tetramer analysis by flow cytometry. Each line represents the frequency of OVA_257-264_ specific, tetramer+ T cells of all CD8 T cells in a single mouse. **B.** Mice were vaccinated 29mer peptide (50 μg) encompassing a known neoantigen[57] and poly(I:C) (10 μg) on days 21–24 or on days 0–3 and 21–24. Splenocytes were harvested on day 28, processed, stimulated with the previously described minimal neoantigen (STHVNHL**H**C) or media alone, and assessed by IFN-γ ELISPOT. One naive mouse was used as a negative control.
(TIF)

S2 Fig Therapeutic vaccination with minimally immunogenic, or cross-reactive mutant peptides. Mice (n = 5 per group) were inoculated with ID8-G7 tumor cells (10^6^ cells/mouse) on day 0 and vaccinated on days 3–6 with individual mutant peptides (50μg) and poly(I:C) (10μg). As a positive control, one group of mice received adoptive transfer of OT-I splenocytes on day 2, followed by vaccination on days 3–6 with ovalbumin (OVA) protein (100 μg) and poly(I:C) (10 μg). Non-vaccinated mice served as negative controls. Each graph represents one group of 5 mice vaccinated with a single mutant 29mer peptide. The same group of positive and negative control mice were used for each graph. Mice were euthanized once they displayed abdominal distension due to ascites.
(TIF)

S1 Table All assessed mutations in the ID8-G7 exome. Table showing each mutation that passed the filtering described in methods.
(XLS)

S2 Table All mutant epitopes with IC50 < 500 nM. Table showing all mutations that passed the filtering described in methods and encoded within peptides predicted to bind H-2Kb or H-2Db with IC50 < 500 nM.
(XLSX)
