# Supplementary figures and images for: Low Mutation Burden in Ovarian Cancer May Limit the Utility of Neoantigen-Targeted Vaccines
Source: PLoS One. 2016 May 18;11(5):e0155189. doi: 10.1371/journal.pone.0155189 (PMC4871527; doi:10.1371/journal.pone.0155189)

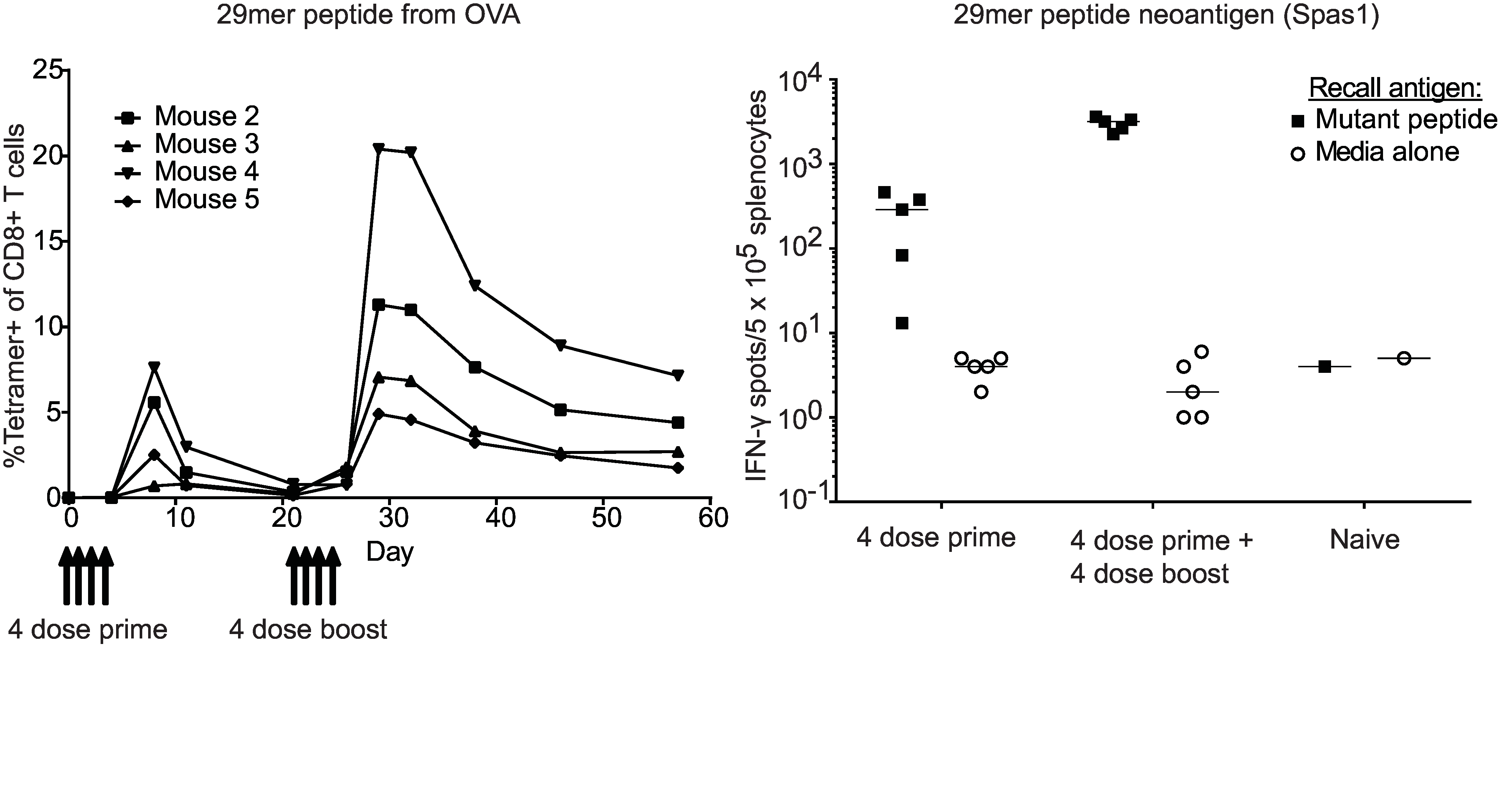

Supplement: S1 Fig — A. Mice were vaccinated with 29mer peptides (50 μg) encompassing the SIINFEKL epitope and poly(I:C) (10 μg) on days 0–3 and 21–24, blood was harvested every 2–4 days, and blood draws were processed for tetramer analysis by flow cytometry. Each line represents the frequency of OVA257-264 specific, tetramer+ T cells of all CD8 T cells in a single mouse. B. Mice were vaccinated 29mer peptide (50 μg) encompassing a known neoantigen[57] and poly(I:C) (10 μg) on days 21–24 or on days 0–3 and 21–24. Splenocytes were harvested on day 28, processed, stimulated with the previously described minimal neoantigen (STHVNHLHC) or media alone, and assessed by IFN-γ ELISPOT. One naive mouse was used as a negative control. (TIF) [file pone.0155189.s001.tif]

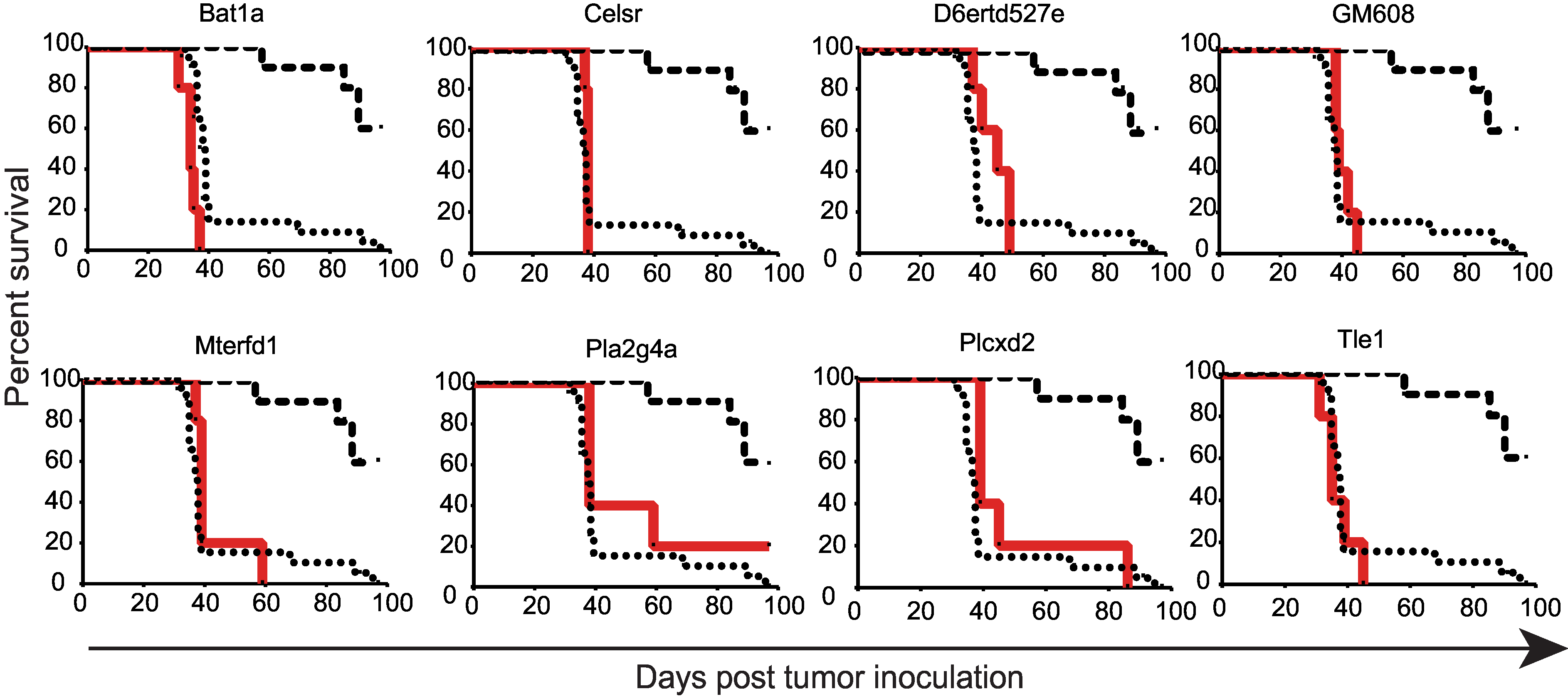

Supplement: S2 Fig — Mice (n = 5 per group) were inoculated with ID8-G7 tumor cells (106 cells/mouse) on day 0 and vaccinated on days 3–6 with individual mutant peptides (50μg) and poly(I:C) (10μg). As a positive control, one group of mice received adoptive transfer of OT-I splenocytes on day 2, followed by vaccination on days 3–6 with ovalbumin (OVA) protein (100 μg) and poly(I:C) (10 μg). Non-vaccinated mice served as negative controls. Each graph represents one group of 5 mice vaccinated with a single mutant 29mer peptide. The same group of positive and negative control mice were used for each graph. Mice were euthanized once they displayed abdominal distension due to ascites. (TIF) [file pone.0155189.s002.tif]
